# A Machine Learning-Based Predictive Model of Epidermal Growth Factor Mutations in Lung Adenocarcinomas

**DOI:** 10.3390/cancers14194664

**Published:** 2022-09-25

**Authors:** Ruimin He, Xiaohua Yang, Tengxiang Li, Yaolin He, Xiaoxue Xie, Qilei Chen, Zijian Zhang, Tingting Cheng

**Affiliations:** 1 School of Nuclear Science and Technology, University of South China, Hengyang 421001, China; 2 Department of Radiation Oncology, The Second Affiliated Hospital, Hengyang Medical School, University of South China, Hengyang 421001, China; 3 Department of Radiation Oncology, Hunan Cancer Hospital, Changsha 410013, China; 4 Department of Computer Science, University of Massachusetts Lowell, Lowell, MA 01854, USA; 5 Xiangya Lung Cancer Center, Xiangya Hospital, Central South University, Changsha 410008, China; 6 Department of Radiation Oncology, Xiangya Hospital, Central South University, Changsha 410008, China; 7 National Clinical Research Center for Geriatric Disorders, Xiangya Hospital, Changsha 410008, China; 8 Department of General Practice, Xiangya Hospital, Central South University, Changsha 410008, China

**Keywords:** machine learning, epidermal growth factor, radiomics, lung adenocarcinoma

## Abstract

**Simple Summary:**

Targeted therapy against epidermal growth factor (EGFR) mutations has become the standard of care for non-small cell lung cancer, and there has not been an efficient genetic test for non-small cell lung cancer patients. The present study aims to find a novel data-driven genetic testing method that can effectively predict the mutation status of EGFR based on a prediction model combining clinical features. The results of this study provide a powerful theoretical basis for the establishment of an effective mutation prediction model. The prediction model can provide a high reference value aiding in EGFR mutation diagnosis and subsequent treatment course.

**Abstract:**

Data from 758 patients with lung adenocarcinoma were retrospectively collected. All patients had undergone computed tomography imaging and EGFR gene testing. Radiomic features were extracted using the medical imaging tool 3D-Slicer and were combined with the clinical features to build a machine learning prediction model. The high-dimensional feature set was screened for optimal feature subsets using principal component analysis (PCA) and the least absolute shrinkage and selection operator (LASSO). Model prediction of EGFR mutation status in the validation group was evaluated using multiple classifiers. We showed that six clinical features and 622 radiomic features were initially collected. Thirty-one radiomic features with non-zero correlation coefficients were obtained by LASSO regression, and 24 features correlated with label values were obtained by PCA. The shared radiomic features determined by these two methods were selected and combined with the clinical features of the respective patient to form a subset of features related to EGFR mutations. The full dataset was partitioned into training and test sets at a ratio of 7:3 using 10-fold cross-validation. The area under the curve (AUC) of the four classifiers with cross-validations was: (1) K-nearest neighbor (AUCmean = 0.83, Acc = 81%); (2) random forest (AUCmean = 0.91, Acc = 83%); (3) LGBM (AUCmean = 0.94, Acc = 88%); and (4) support vector machine (AUCmean = 0.79, Acc = 83%). In summary, the subset of radiographic and clinical features selected by feature engineering effectively predicted the EGFR mutation status of this NSCLC patient cohort.

## 1. Introduction

According to statistics by the World Health Organization (WHO), lung cancer has become the leading cause of cancer-related deaths worldwide. Lung cancer can be categorized into small cell lung cancer and non-small cell lung cancer (NSCLC), where NSCLC accounts for 80–85% of all lung cancer cases [1]. The major treatment modalities for lung cancer include surgical resection, postoperative chemotherapy, radiotherapy, or, if surgical resection is not feasible, then combination therapy, depending on the diagnostic stage. Several retrospective studies have discovered [2,3,4] that patients with NSCLC harbor different driver mutations (EGFR, BRAF, ROS1, MET, ALK, etc.), among which epidermal growth factor receptor (EGFR) mutations are prevalent. In the past decade, molecular translational research has led to tremendous breakthroughs in cancer diagnosis and treatment, particularly in the development of new targeted therapies against key signaling pathways involved in the malignant progression of lung cancer [5,6]. EGFR-specific small-molecule tyrosine kinase inhibitors (TKIs) were the first targeted agents to enter the standard treatment regimen for NSCLC. Clinical trials have shown [7,8,9] that patients positive for EGFR mutations treated with targeted TKI agents (erlotinib, afatinib, etc.) have improved progression-free survival (PFS) and treatment tolerability compared to other first-line chemotherapy regimens, whereas in patients with wild-type EGFR, PFS is shorter with targeted agents such as gefitinib compared to chemotherapy regimens using platinum-based agents. Therefore, it is of great clinical importance to rapidly and accurately identify the EGFR mutation status in NSCLC patients.

A previous epidemiological study [10] has shown that several clinical factors (female, no smoking history, adenocarcinoma, and East Asian ethnicity) are associated with a high prevalence of EGFR mutations; however, there are no robustly predictive clinical features of EGFR mutation status. Thus, sequencing-based mutation detection remains the gold standard for identifying mutations in NSCLC patients. Although sequencing is the most basic and straightforward method for genetic testing [11,12], it is a cumbersome and inherently insensitive process that is not suitable for the analysis of large numbers of clinical samples. Compared with sequencing, other molecular biology methods, although with improved sensitivity and specificity, are only effective for some common mutations. In addition to the characteristics of the assay itself, the process of genetic testing with biopsy specimens is affected by many other factors, such as sample quality, sample content, pathology type, and degree of differentiation, so there is a great need for a rapid, easy, cost-effective, and accurate mutation detection method.

Computed tomography (CT) is the primary imaging tool used for the diagnosis of lung cancer in the current clinical workflow. Therefore, extracting relevant information from these routine images is inherently high yield. There is currently no evidence linking EGFR status in NSCLC patients solely to CT image features; however, the latest radiomic methods [13,14] have provided a quantitative analysis of tumors and their microenvironment by extracting minable high-content data features to establish imaging prediction models. The application of these radiomic methods has many advantages. For example, not only can they provide an effective clinical prediction for patients who are not eligible for biopsy, but their successful application would also provide additional reference information for EGFR-negative patients. In this study, we proposed a machine learning-based method to identify imaging biomarkers of EGFR mutations in domestic NSCLC patients through imaging analysis.

## 2. Materials and Methods

### 2.1. Patient Data

All patient data were retrospectively collected from patients diagnosed with lung cancer between July 2013 and February 2022 where EGFR status was determined following surgery or pathological biopsy at Xiangya Hospital, Hunan Cancer Hospital, or the Second Hospital, the University of South China. A total of 758 cases were enrolled according to the inclusion criteria, with a median age of 55.6 (range 23–85) years. Of the enrolled cases, 396 cases were EGFR wildtype (EGFR−)and 362 cases harbored EGFR mutations (EGFR+). The final data were divided into training and validation groups at a 7:3 ratio. A total of 530 patients (69.9%, 246 EGFR+, and 284 EGFR−) from Hunan Cancer Hospital were used as the training set, and 228 patients (30.1%, 116 EGFR+, and 112 EGFR−) from Xiangya Hospital of Central South University and the Second Affiliated Hospital of South China University were used as the external validation dataset. The current study was approved by the hospital ethics committee, thereby waiving the informed consent of patients.

### 2.2. Case Selection

The inclusion criteria were: (1) preoperative images that could be completely read; (2) the interval between CT examination and pathological biopsy was not more than three months; and (3) no preoperative treatment of any kind. The exclusion criteria were: (1) preoperative CT images with large artifacts or poor image quality; (2) preoperative neoadjuvant chemotherapy; and (3) no EGFR gene test results.

### 2.3. Patient Characteristics

Clinical data of the patients gathered included gender, date of diagnosis, age, smoking history, pathological stage, and family history. The smoking history statistic was divided into smokers and non-smokers; EGFR mutation status was divided into unmutated and mutated groups (exon 15–21 mutation), and staging was divided into groups I–II and III–IV. The pathological staging of the tumors followed the NCCN Clinical Practice Guidelines for Non-Small Cell Lung Cancer version 2. 2022. The statistical summary of the enrolled patients in this study is shown in Table 1.

### 2.4. CT Examination

All patients were scanned in the flat scan enhancement mode with either a GE Light speed 16 (GE Medical Systems, Milwaukee, WI, USA), GE Discovery CT750 (GE Medical Systems, Milwaukee, WI, USA), or a Brilliance iCT (Philips Medical Systems, Cleveland, OH, USA) with the following acquisition parameters: tube voltage of 120 kV, tube current of 150–200 mA, scan layer thickness of 5 mm, and reconstruction thickness and interval of 1.5–3 mm. All image data (before extracting features) were aligned using the Elastix module (version 5.0.1, Linux Foundation, San Francisco, CA, USA, https://elastix.lumc.nl, accessed on 20 July 2021) in 3D-Slicer, and the images were batch pixel standardized and normalized using specific scripts in Jupyter Notebook.

### 2.5. Region of Interest (ROI) Labeling

**Figure 1:**
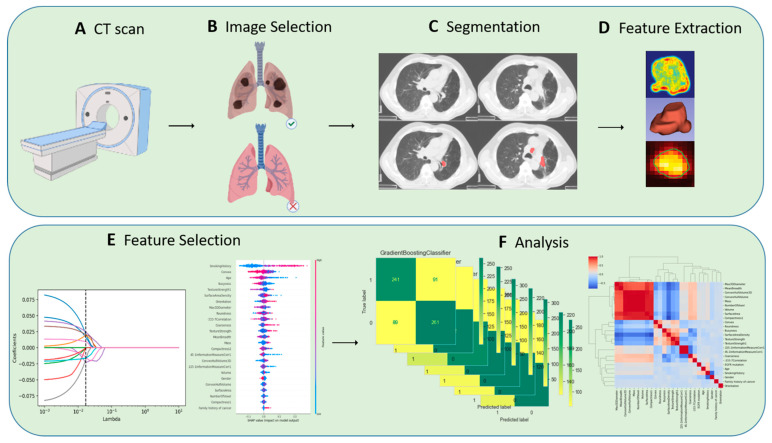
Overview of radiomics workflow.

The image analysis platform 3D Slicer (version 4.11, https://www.slicer.org/, accessed on 15 August 2021) was used in this study. The ROI segmentation was performed by two radiologists from the thoracic and abdominal groups, both with more than five years of relevant experience. The segmentation method was semi-automated with the following workflow: pre-processing, semi-automatic correction of lung boundaries, solid and ground glass shadow boundary processing, manual refinement editing, etc. Images segmented by two of the radiologic imaging specialists were selected for intraclass correlation coefficient (ICC) consistency review, and discrepancies were resolved through discussion until consensus was reached. The overview of the radiomics workflow is illustrated in Figure 1.

### 2.6. Feature Engineering

SlicerRadiomics™ (version 2.10, http://github.com/Radiomics/SlicerRadiomics, accessed on 10 December 2021) was employed in this work. This is a scripted loadable module bundled in the 3D Slicer extension. It gives access to the radiomics feature calculation classes implemented in pyradiomics library. 3D Slicer was used to extract a total number of radiomic features. These features can be divided into three categories: (1) texture-based, (2) shape-based, and (3) intensity histogram-based. Texture features include Gray-level co-occurrence matrix (GLCM), Gray-gradient co-occurrence matrix (GGCM), Gray-level run-length matrix (GLRLM), Neighborhood-Intensity-Difference (NID), etc. Shape-based features include compactness, volume, surface area, Max3Ddiameter, etc. Intensity includes histogram kurtosis, energy, entropy, etc. To improve the model’s generalization ability and fitting efficiency, redundant features were deleted and feature repeatability and stability were tested. Two alternative feature selection and dimensionality reduction strategies were used to choose a subset of features with acceptable reproducibility.

### 2.7. Feature Selection and Modeling

The least absolute shrinkage and selection operator is a regularization-based algorithm. Unlike ridge regression, the LASSO algorithm adds the L1 norms to the cost function of standard linear regression as the penalty function and updates the value of the weight coefficient λ by iteration until the optimal solution is found; the insignificant feature weight coefficients are compressed to zero, thus achieving the purpose of feature selection. The least absolute shrinkage and selection operator (LASSO) was used to filter the features using logistic regression in order to generate a regression function which kept features with non-zero coefficients to form a subset of high discrimination features. Principal component analysis (PCA) and the Shapley value algorithm were also applied to downscale and filter the radiomic features. Concordant features among the two screening methods were selected for predictive modeling.

### 2.8. Application of Shapley Value Algorithm to PCA

The feature vector corresponding to the first n feature values containing more than 95 percent of the information was maintained, and the maximum variance value was determined. However, because the features processed by PCA after dimensionality reduction are difficult to interpret, the game theory-based Shapley (Shapley value explanation) value algorithm [15] was used to calculate and rank the importance of the imaging features, with only the top n features being kept. The general steps of the algorithm implementation are: (1) obtain the subset S of features that do not contain X (i); (2) predict the effect after adding X (i) to all feature subsets separately; and (3) calculate the marginal contribution value of feature X (i) by summing all results. The predictive power of feature X (i) for label values is weighted by all possible marginal contribution values. The formula for the weight of feature X (i) is expressed as follows:

$$\emptyset_{i}^{x}=\frac{1}{N}\underset{S\subseteq N\backslash \left\{i\right\}}{\sum }{\left(\frac{N-1}{S}\right)}^{-1}\left(v_{x}\left(S\cup \left\{i\right\}\right)-v_{x}\left(S\right)\right)$$

where ***N*** is the number of all features, ***S*** is the feature subset consisting of S features, and ***v_x_*** represents an importance parameter mapped from the subset.

### 2.9. Model Establishment and Statistics Analysis

All statistical analyses and modeling efforts were performed using Jupyter notebook (version 6.1.4, https://jupyter.org/, accessed on 13 June 2021) and SPSS (IBM, Armonk, NY, USA, version 20.0), compiled in Anaconda (version 1.10.0, Broadway Ave, NY, USA, https://www.anaconda.com, accessed on 5 June 2021). For continuous and categorical variables of clinical and pathological characteristics, one-way *t*-tests, chi-square tests, and multiple logistic regression analyses were conducted, and two-sided *p* values < 0.05 were considered statistically significant. The screened feature subsets were subjected to machine learning model construction, and the training and test sets were randomly assigned in a 7:3 ratio. The K-fold method was used for ten-fold cross-validation. The training set was trained and validated using Random Forrest (RF), K-nearest neighbor (KNN), Light Gradient boosting (LGBM), and Support vector machine (SVC), respectively. The validation set uses the receiver operating curve (ROC) and the area under the curve (AUC) to evaluate the classification effect of the model.

## 3. Results

### 3.1. Clinical Characteristics Statistics Analysis

Demographic and pathological data of the patients are listed in Table 2. All enrolled cases were diagnosed as NSCLC with a median age of 55.6 ± 10 (23–85) years. The pathological stages were as follows: 602 patients were stage IV (79.4%), 71 patients were stage IIIA (9%), 55 patients were stage IIIB (7.2%), five patients were stage IIIC (0.7%), and seven patients were stage IIA and IIB. A total of 362 cases presented with mutated EGFR (exon 15–21), and 396 cases harbored wild-type EGFR. The EGFR mutation status was found to be statistically significantly different by gender and smoking history (*p* < 0.001).

### 3.2. Radiomics Features Selection

**Figure 2:**
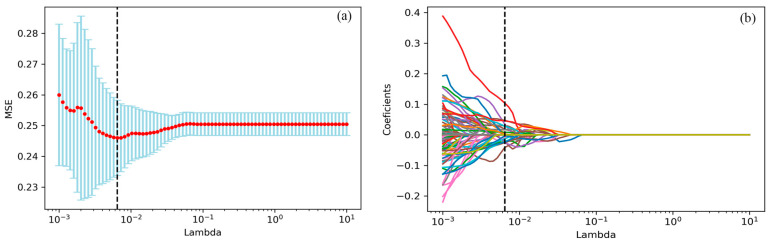
the LASSO algorithm for features selection. (**a**) The mean square error path using ten-fold cross-validation. (**b**) LASSO coefficient profile of 622 features, where 31 features with non-zero coefficients were selected.

**Figure 3:**
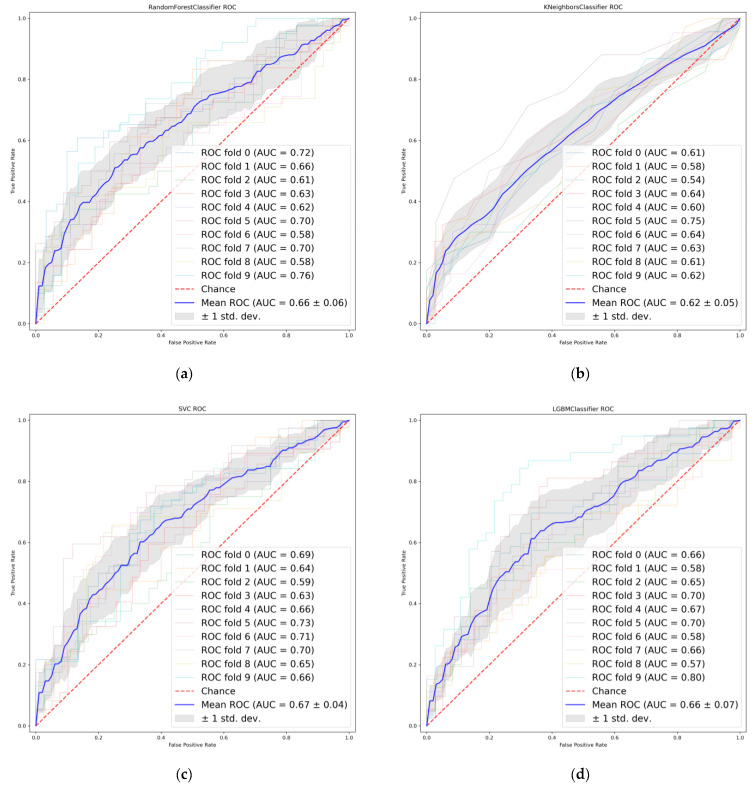
Receiver operating characteristics (ROC) curves of LASSO features. (**a**) Random Forest. (**b**) K-nearest neighbor. (**c**) Support vector machine. (**d**) Light Gradient boosting.

Thirty-one radiomic features with non-zero penalty function coefficients were selected by the LASSO regression classifier (Figure 2) from a total of 622 features, and a subset of the screened features (Table 3) was selected for EGFR mutation status prediction modeling based on the 31 feature subsets selected by LASSO regression. The predictive model results are shown in Figure 3, and the AUCmean for the RF model is 0.66 ± 0.06; the AUCmean for the KNN is 0.62 ± 0.05; the AUCmean for the SVC = 0.67 ± 0.04; and the AUCmean for the LGBM model = 0.66 ± 0.07. The test set accuracies are: RF Acc = 61%; KNN Acc = 57%; SVC Acc = 60%; and LGBM Acc = 61%. The performance of the classifier on the training and test sets and the F1 scores are shown in Table 4.

**Figure 4:**
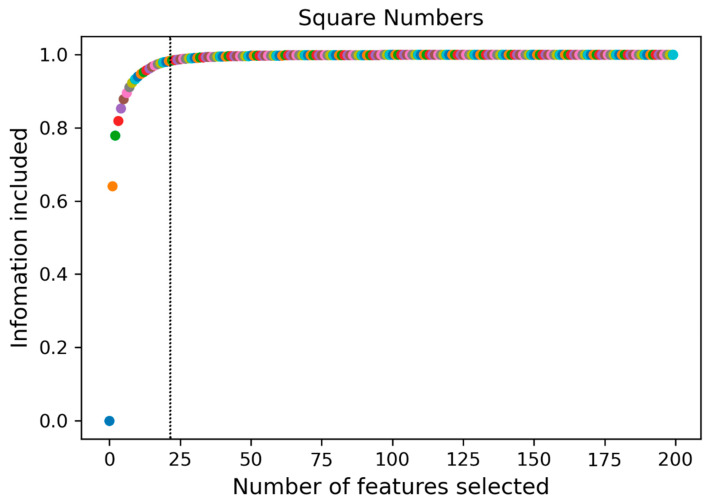
A subset of 24 features containing 95% of the principal components was obtained by principal component analysis (PCA).

The radiomic feature values were normalized to be between [−1, 1] by PCA, with all metrics approximating a normal distribution. The features with the top 24 variance percentages contained more than 95% of the information of the original radiomic features (Figure 4), and this feature subset was set as the principal component of the original sample (Table 5).

**Figure 5:**
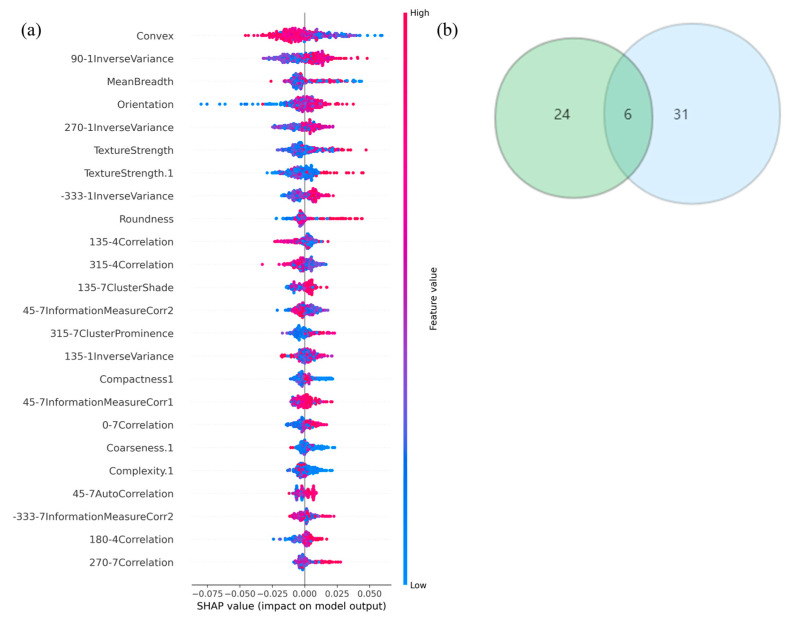
(**a**) The Shapely-value ranking of every single feature. (**b**) Six common radiomics features between PCA and LASSO features, respectively.

The ranking of feature importance in the subset was derived from the Shapley value algorithm (Figure 5a), The top five radiomics features in terms of predictive power for EGFR mutation status were (1) Convex, (2) 90-1InverseVariance, (3) MeanBreadth, (4) Orientation, and (5) TextureStrength.

**Figure 6:**
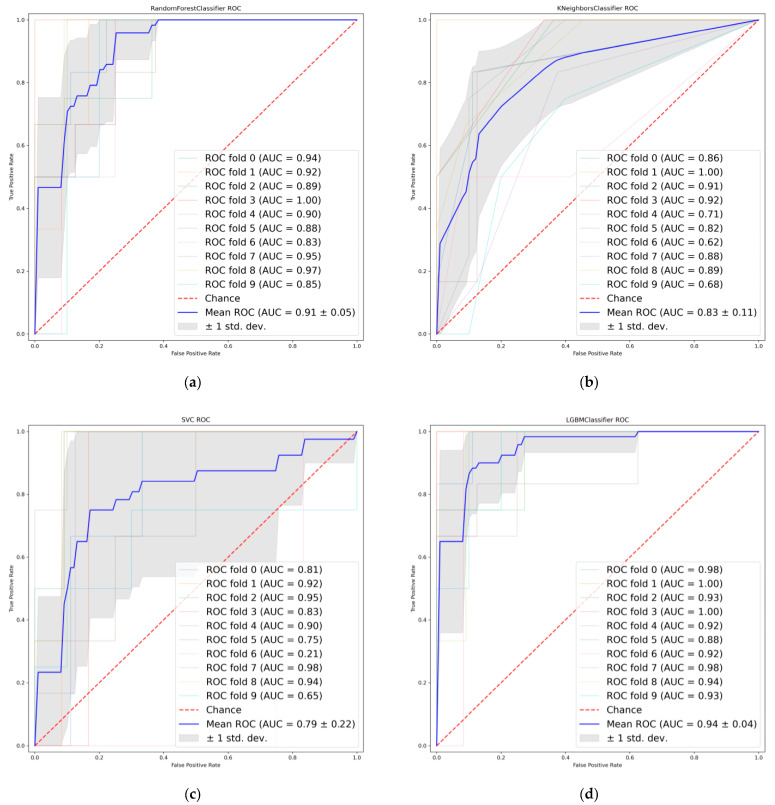
Receiver operating characteristics (ROC) curves when using the combined features. (**a**) Random Forest. (**b**) K-nearest neighbor. (**c**) Support vector machine. (**d**) Light Gradient boosting.

Six radiomic features with good repeatability were found between the LASSO and PCA-Shapley methods (Figure 5b). These six features were combined with four clinical features to obtain a subset of radiomic and clinical features (Table 6). The predictive model was established and evaluated again, and the dataset was split into a training set and a test set (7:3). Ten-fold cross-validation was performed on the test data using four classifiers (Figure 6), in which the average AUC value of the RF model was 0.91, the average AUC value of the KNN model was 0.83, and the average AUC value of the SVC model was 0.79. The average AUC value for the LGBM model was 0.94. The test set accuracies were: RF Acc = 83%; KNN Acc = 81%; SVC Acc = 83%; and LGBM model LGBM Acc = 88%. In the same way, the performance of the classifier on the training and test sets and the F1 scores of the new model are shown in Table 7.

## 4. Discussion

A variety of methods have been developed to detect EGFR mutations, such as the polymerase chain reaction amplification gene direct sequencing method, high-resolution lysis analysis, and fragmentation analysis [16,17]. However, each of these methods require an invasive pathological biopsy to extract tumor samples, which is not only costly and poorly reproducible but can also yield false-negative results. The aim of our current study was to establish a non-invasive, novel genetic detection method via the correlation of radiomic features to the standard genetic detection of EGFR status in NSCLC tumors.

In this study, EGFR mutations were found in 47.8% of all patients. The most common mutations of exon 19 and exon 21 (28.8% and 15.2%, respectively) are consistent with the results of published studies of relevant Asian patients [18,19,20]. There were also rarer cases: one patient with an exon 15 mutation and 12 patients with confirmed double mutations in exon 20 (TKI-resistant mutation) and exon 21 (TKI-sensitive mutation). Several studies [18,21] have shown that EGFR mutations are mostly seen in patients with lung adenocarcinoma without a smoking history, that female, of the peripheral type, and are not associated with factors such as age and pathological stage, which are further validated in our current study. Given the high rate of EGFR mutations in Chinese lung adenocarcinoma patients, a previous study [22] has demonstrated that exon 19 and 21 mutations combined with clinicopathological features could be a molecular marker to assess the efficacy of TKI treatment for NSCLC. Therefore, it is necessary to explore reliable evidence to predict EGFR mutations in addition to clinical and pathological factors. Several studies [23,24,25] have shown that radiomic features can quantify the overall tumor and peripheral microenvironment, reflecting different gene expression types. Moreover, additional studies [26,27] have also pointed out that the EGFR mutation status is related to gender smoking history and histological subtype (squamous cell adenocarcinoma), which is consistent with the results of this study. Furthermore, previous studies [26,28,29] were based on machine-learning using hand-draft features, and the effect of the model combined with clinical features was better than that of the single radiomics features model or clinical features, but the prediction effect of the combined features subset in these studies achieved about Auc 0.8. However, our current study not only coupled radiomic and clinical features but also used different screening methods to obtain feature subsets with good repeatability in the feature selection stage, which has greatly improved the modeling effect. Among the four classifiers, the LGBM classifier was found to offer the best effect (AUCmean = 0.94, acc = 88%). Despite the advantages of machine-learning-based radiomics methods, hand-draft features require time-consuming boundary segmentation of the lesioned tissue, so some studies [24,25,30] have proposed the use of deep learning methods to learn certain gene-related features, thus avoiding the four complex procedures of features engineering. On the other hand, our objective can be further clarified in that although the mutation status is significant for the decision of patient treatment, it is more clinically relevant for personalized treatment if the subtypes of mutation can be distinguished. Mutations in the EGFR and echinoderm microtubule-associated protein 4-mesenchymal lymphoma kinase fusion (EML4-ALK) mutations are more common in NSCLC, so in future research we will analyze the mutation types and subtypes of these two genes and introduce survival analysis of combination therapy and prognosis analysis of targeted therapy.

Six radiomics features with relevance to the mutation were extracted, including four shape-based features (Convex, MeanBreadth, Orientation, Compactness1) and two texture-based features (TextureStrength, Correlation). Shape-based features are very significant descriptors of heterogeneity and visual intuition of the tumor, while texture-based features are defined as a repetitive arrangement of some basic pattern of the image [31]. Some researchers [32] proposed in their studies for the first time that texture-based features of medical images can be used to predict the effects of tumor treatment. The same shape features have also been used in several studies [33,34] to build predictive and prognostic models for certain specific diseases. In summary, the subset of features in the study have potential as biomarkers.

Generally, with the development of medical imaging data and sophisticated image analysis tools, radiomics has gradually matured to provide an effective decision-making tool for personalized treatment plans in modern medicine [35]. It should be noted that there are several limitations to our study. First, this study is a retrospective study with some positive and negative sample imbalances between groups, which could affect the results of the analysis of variables. Second, multi-center data were used. Although this study considered the issue of feature reproducibility in the feature screening process, the brands and the parameters of scanning machines were inconsistent during the data collection phase, and ROIs were all outlined semi-automatically by radiologists, which could affect the reproducibility and stability of radiomic features. In future studies, we will establish improved quality evaluation criteria to further promote the standardized assessment and relevance of radiomics methods to clinical problems. Finally, although the model test results based on feature subsets achieved high accuracy, it is still worth exploring whether the feature selection method combining PCA and Shapley values in this study was reasonable. We will continue to optimize the sample structure in future studies or conduct prospective studies based on more homogeneous patient samples to strengthen the reliability and stability of the model for gene mutation prediction so as to provide personalized treatment plans for lung cancer patients.

## 5. Conclusions

We have developed a machine learning-based method for identifying the status of EGFR mutations in NSCLC which can provide radiologists with a quantitative and intuitive method to determine the type of gene mutation in lung cancer. Although the results were not satisfactory in the initial training stage, better prediction results were achieved by a random distribution of positive and negative samples, the adjustment of hyperparameters of classifiers, and the optimization of overfitting. Our results indicate that this method has a great potential application for gene expression prediction. Nonetheless, it is still necessary to implement valid external validation in combination with additional multi-center data to improve the stability and reliability of our method. We have also highlighted the feasibility of non-invasively detecting the EGFR genetic status in lung adenocarcinomas using a machine learning model based on combined CT radiomic features and clinical characteristics. Hopefully it can provide additional information for the treatment strategy of lung adenocarcinoma patients.

## Figures and Tables

**Table 1:** Clinical characteristics of patients.

Characteristics	Groups	Overall	
Gender	Male	441	58.2%
	Female	317	41.8%
Smoking History	Yes	358	47.2%
	No	400	52.8%
Stage	IV	602	79.4%
	IIIA	71	9%
	IIIB	55	7.2%
	IIA	7	0.9%
	IIIC	5	0.7%
	IB	6	0.8%
	IIB	6	0.8%
	IA	4	0.5%
	II	2	0.3%
Family History	Yes	79	10.4%
	No	679	89.6%
Tumor Location	Central Type	239	31.5%
	Peripheral Type	513	67.7%
	unknown	6	0.8%
EGFR Mutation	wild-type	396	52.2%
	exon 19	218	28.8%
	exon 21	115	15.2%
	exon 20.21	12	1.6%
	exon 18	6	0.8%
	exon 19.20	5	0.7%
	exon 19.21	3	0.4%
	exon 18.20.21	1	0.1%
	exon 18.20	1	0.1%
	exon 15	1	0.1%

**Table 2:** Patients’ baseline characteristics.

Variables		Overall	Mutation	Wild-Type	*p*-Value
Median Age (Range)		55.6 ± 10 (23–85)	55.2 ± 9.9 (23–85)	55.9 ± 10.1 (29–83)	0.34
Gender	Female	317	188	129	<0.001
	Male	441	174	267	
Smoking History	No	400	236	164	<0.001
	Yes	358	126	232	
Family History	No	679	326	353	0.633
	Yes	79	36	43	
Stage	I–II	25	9	16	0.309
	III–IV	733	352	381	
Tumor Location	C-Type	239	110	129	0.583
	P-Type	513	248	265	

**Table 3:** Feature selection by LASSO regression. (Top 10).

Groups	Feature Names	Coefficients
Shape	Convex	−0.02970268
	Orientation	0.00904001
	MeanBreadth	−0.0272518
GLCM	-333-4Correlation	−0.0582105
	135-4InformationMeasureCorr1	−0.01293069
	45-7SumVariance	0.005674244
	135-7ClusterTendendcy	0.00998332
	90-7DifferenceEntropy	−0.02322079
	0-4InverseVariance	0.06418816
NID	Busyness	0.03406641

**Table 4:** Predictive performance of four models.

Model Name	Train ROC/ AUC Mean	Test ROC/ AUC Mean	*p*	Train Acc Mean	Test Acc Mean	*p*	Train F1 Mean	Test F1 Mean	*p*
LGBM	0.99	0.64	0.03	0.95	0.61	0.03	0.95	0.63	0.02
RF	1	0.65	0.03	1	0.61	0.02	1	0.62	0.04
SVC	0.72	0.65	0.03	0.64	0.6	0.01	0.65	0.61	0.01
KNN	0.76	0.58	0.01	0.7	0.57	0.02	0.7	0.57	0.02

**Table 5:** Features reduction by PCA (Top 10).

Groups	Feature Names
Shape	Roundness
	Convex
	Orientation
	MeanBreadth
NID	TextureStrength1
	TextureStrength
GLCM	90-1InverseVariance
	270-1InverseVariance
	-333-1InverseVariance
	135-4Correlation

**Table 6:** A combined subset of features with radiomics and clinical.

Feature Type	Feature Name
Radiomics	Convex
	Meanbreadth
	Orientation
	TextureStrength
	Compactness1
	270-7Correlation
Clinical	SmokingHistory
	Age
	Gender
	FamilyHistory

**Table 7:** The performance of four predictive models using the combined features.

Model Name	Train ROC/ AUC Mean	Test ROC/ AUC Mean	*p*	Train Acc Mean	Test Acc Mean	*p*	Train F1 Mean	Test F1 Mean	*p*
LGBM	1	0.926	0.084	1	0.88	0.11	1	0.72	0.32
RF	1	0.91	0.079	1	0.832	0.1	1	0.66	0.28
SVC	0.897	0.87	0.09	0.87	0.831	0.12	0.75	0.64	0.22
KNN	0.947	0.84	0.096	0.9	0.81	0.09	0.79	0.63	0.18

## Data Availability

The data in this research are available upon request.
